# Age- and Sex-Specific Standard Scores for the Reading the Mind in the Eyes Test

**DOI:** 10.3389/fnagi.2020.607107

**Published:** 2021-01-28

**Authors:** Jana Kynast, Maryna Polyakova, Eva Maria Quinque, Andreas Hinz, Arno Villringer, Matthias L. Schroeter

**Affiliations:** ^1^Department of Neurology, Max Planck Institute for Human Cognitive and Brain Sciences, Leipzig, Germany; ^2^LIFE - Leipzig Research Centre for Civilization Diseases, University of Leipzig, Leipzig, Germany; ^3^Clinic for Cognitive Neurology, University Hospital Leipzig, Leipzig, Germany; ^4^Department of Medical Psychology and Sociology, University Hospital Leipzig, Leipzig, Germany

**Keywords:** theory of mind, mindreading, Reading the Mind in the Eyes test, social cognition, neuropsychological testing, cognitive aging, dementia

## Abstract

The reliable, valid and economic assessment of social cognition is more relevant than ever in the field of clinical psychology. Theory of Mind is one of the most important socio-cognitive abilities but standardized assessment instruments for adults are rare. The Reading the Mind in the Eyes Test (RMET) is well-established and captures the ability to identify mental states from gaze. Here, we computed standard scores for the German version of the RMET derived from a large, community-dwelling sample of healthy adults (20–79 years). The standardization sample contains 966 healthy adult individuals of the population-based Leipzig Research Center for Civilization Diseases (LIFE) study. Before standardization, weighting factors were applied to match the current sample with distribution characteristics of the German population regarding age, sex, and education. RMET scores were translated into percentage ranks for men and women of five age groups (20–29, 30–39, 40–49, 50–59, 60+ years). Age-specific percentage ranks are provided for men and women. Independent of age, men present a larger variance in test scores compared to women. Within the specific age groups, women score higher and their scoring range is less variable. With increasing age, the scoring variance increases in both men and women. This is the first study providing age- and sex-specific RMET standard scores. Data was weighted to match German population characteristics, enabling the application of standard scores across German-speaking areas. Our results contribute to the standardized assessment of socio-cognitive abilities in clinical diagnostics.

## Introduction

Social cognition is one of the most complex and unresolved concepts in psychology and neurosciences. It encompasses numerous definitions (Happé et al., 2017) and includes many socio-cognitive abilities. The ability to identify mental states, intentions, desires, feelings and beliefs of self and others (Premack and Woodruff, 1978) is called Theory of Mind (ToM). It comprises the perception, processing and integration as well as the interpretation of social context information facilitating the prediction of another individual's future actions and the adaptation of own behavioral responses. Thus, ToM contributes to successful social interactions. Contrary, impairment in ToM can compromise the initiation and maintenance of positive relationships. Among various psychiatric disorders and neurological diseases, brain injury, age-related brain changes, as well as developmental and neurodegenerative disorders (e.g., Gregory et al., 2002; Brune and Brune-Cohrs, 2006; Schroeter et al., 2010, 2014; Schroeter, 2012; Kynast et al., 2018; Vallat-Azouvi et al., 2018) have been characterized by ToM deficits. Further, the clinical relevance of ToM increased recently, when the Diagnostic Statistical Manual of Mental Disorders' 5th edition (DSM-5; American Psychiatric Association, 2013) explicitly defined social cognition as one key domain for neuropsychological assessment, especially in the context of acquired cognitive dysfunction, i.e., major and mild neurocognitive disorder (NCD; corresponding terms are dementia and its prestage, i.e., mild cognitive impairment, MCI).

Standardized psychological tests help to identify specific deficits (and resources) as they provide normative scores for the classification of individual abilities relative to a reference group (ideally) age-, sex- and education-matched. Performance deviations might indicate the need for subsequent in-depth testing to further evaluate and confirm the detected deficits as well as to classify potential impairment regarding activities of daily living. However, the systematic assessment of socio-cognitive abilities, particularly ToM, is problematic, as only a few instruments are available for adults and even fewer are standardized.

The *Reading the Mind in the Eyes Test* (RMET; Baron-Cohen et al., 1997, 2001) may be a potential candidate for standardized assessment of specific aspects of social cognition. It captures the ability to identify mental states based on the eyes of another person. The revised version (Baron-Cohen et al., 2001) includes 36 photographs of the eye region of 18 men and 18 women. Each item comes along with four response options. The term most appropriately describing the mental state of the pictured person shall be selected. The RMET requires the identification of complex mental states where social cues are limited (Baron-Cohen et al., 1997). The task addresses especially the identification of the relevant mental state of the stimulus (first stage of ToM attribution) rather than inferring the content of that mental state (second stage; see Baron-Cohen et al., 2001). It is supposed to capture predominantly “hot” or “affective” ToM, since it requires an understanding of another person's affective states or feelings (c.f. “cold” or “cognitive” ToM: a concept of cognitive states, beliefs, thoughts, or intentions; Henry et al., 2013; El Haj et al., 2015). This might be in line with a recent critical note on the validity of the RMET (Oakley et al., 2016), suggesting that the focus of the RMET is rather emotion recognition than ToM as compared to other assessment instruments using cartoons or short movies of social situations. Overall, studies show mixed results regarding the validity of the test (see Olderbak et al., 2015 for review of the psychometric properties; critical aspects regarding test construction can be found in Kynast and Schroeter, 2018). Besides these critical points, the RMET may still be a promising candidate for further clinical application since it has been shown to identify deficits in mental state recognition in psychopathology and neurodegenerative diseases such as behavioral variant frontotemporal dementia (Pardini et al., 2013; Schroeter et al., 2018), Alzheimer's disease and mild NCD (see Baglio et al., 2012), autism (Baron-Cohen et al., 1997, 2015), bipolar disorder (Bora et al., 2005) and small vessel disease (Kynast et al., 2018). The test is not characterized by a ceiling effect since the response rate of healthy individuals is typically below 100% (Pardini and Nichelli, 2009). Furthermore, it is one of the most frequently used tasks to investigate ToM in adulthood, published in more than 250 (Kirkland et al., 2013) or even 542 studies according to PubMed until 6th of April 2020 (term = reading + the + mind + in + the + eyes). Test performance is modulated by individual characteristics such as sex, and age, and the characteristics of the stimulus material itself (e.g., Baron-Cohen et al., 1997; Bailey et al., 2008; Pardini and Nichelli, 2009; Castelli et al., 2010; Cabinio et al., 2015; El Haj et al., 2015; Fischer et al., 2016; Kynast and Schroeter, 2018; Kynast et al., 2018, 2020). To our best knowledge, no standard scores are yet available in any language.

This study is the first to present age-standardized scores for the RMET derived from a large, population-based sample of healthy adults (Loeffler et al., 2015). We used the German version of the RMET (Bölte, 2005). Weighting factors were applied to match the current sample to the characteristic distribution of age, sex and education represented in the German population. RMET scores were transformed into percentile ranks that characterize the performance of an individual in relation to its reference group. The study aimed overall at yielding norms for neuropsychological characterization of adults and potentially identifying deviations due to disease.

## Methods

### Study Cohort

This study was part of the adult study of the Leipzig Research Center for Civilization Diseases (LIFE). The LIFE adult study is a cross-sectional study investigating “prevalences, early onset markers, genetic predispositions, and the role of lifestyle factors of major civilization diseases” (Loeffler et al., 2015). The study was approved by the ethics committees of the University of Leipzig and was conducted in accordance with the latest version of the Declaration of Helsinki. Each subject provided written informed consent. All participants were randomly selected residents of the city of Leipzig (Saxony/Germany, population ~600,000, Amt für Statistik und Wahlen Leipzig, 2020) aged 18–79 years. Participation was voluntary. The total sample comprised 10,000 individuals of whom 2,600 completed structural and functional brain magnetic resonance imaging (MRI). Adults aged 60–79 years completed an in-depth-assessment (neuropsychological testing, medical examinations, interviews on individual lifestyle conditions). Persons younger than 60 years completed a less extensive test battery. The complete protocol is described elsewhere (Loeffler et al., 2015).

For the current study, participants who fulfilled at least one of the following criteria were excluded from data analysis: (1) history of neurological or psychiatric disorder (i.e., dementia, alcohol or substance abuse, schizophrenia, affective and anxiety disorders, eating disorders, autism), (2) intake of medication active on the central nervous system (opioids, hypnotics and sedatives, anti-parkinsonian drugs, anxiolytics, antipsychotics, anti-epileptic drugs), (3) depression score >20 on the Center of Epidemiologic Studies Depression Scale (Radloff, 1977), (4) history of stroke, brain injury or tumor, (5) significant white matter hyperintensities (Fazekas stages 2 and 3) on T2-weighted fluid attenuated inversion recovery (FLAIR) MRI scans (Fazekas et al., 1987). The latter has been selected as a specific exclusion criterion since age-related changes in the brain's white matter structure are associated with cognitive impairment. Especially structural alterations as severe as Fazekas stages 2 and 3 have been associated with significant cognitive deficits in attention, memory, and most crucially, social cognition compared to individuals without white matter hyperintensities (Kynast et al., 2018).

The final standardization sample included 966 adults (48.6% male) aged 20–79 years (mean, *M* = 50.7; standard deviation, *SD* = 16.2). Demographic characteristics, self-report on medical history and medication intake, as well as brain MRI was available for the whole sample. Note that all individuals had normal or corrected to normal vision.

### Test and Procedure

**Figure 1:**
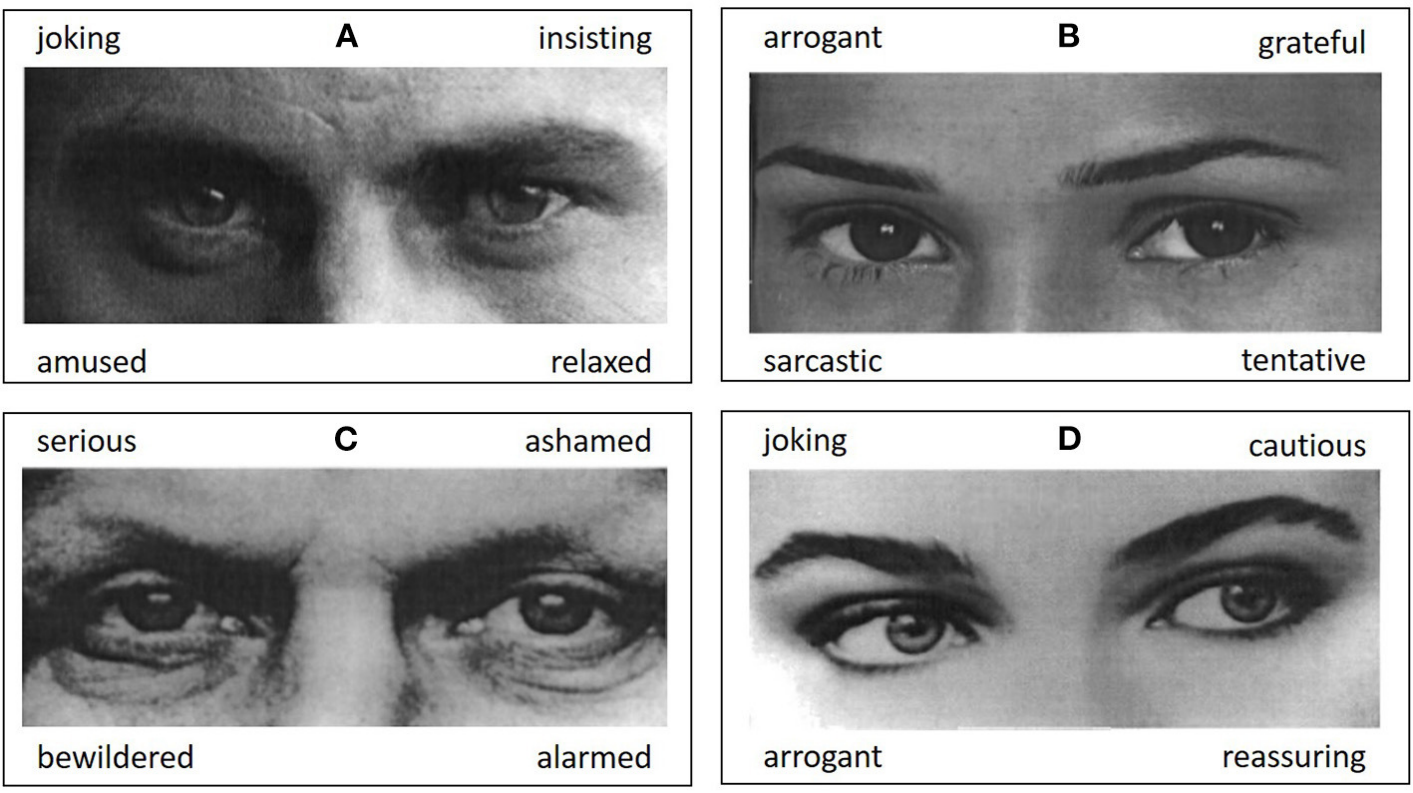
Figure shows example items from the Reading the Mind in the Eyes Test (Baron-Cohen et al., 2001; Bölte, 2005). Out of four response options the most appropriate mental state term shall be selected. Correct responses are **(A)** insisting, **(B)** tentative, **(C)** serious, **(D)** cautious. Pictures are taken from Bölte (2005).

The ability to identify mental states from gaze was assessed with the German version of the revised RMET (Baron-Cohen et al., 2001; Bölte, 2005). The test contains 36 black-and-white photographs of the eye region of either a man or a woman. Each item is presented with four response options of which the word best describing the pictured mental state shall be selected (see Figure 1 for examples). Computerized assessment included standard instructions and a test item (Bölte, 2005). If necessary, trained study assistants provided help with the handling of the hardware for response selection. Assessment was self-paced and needed approximately 10-15 min for completion. Prior to analysis, data was carefully checked for plausibility, i.e., possible biases in response profiles as well as accuracy rates below chance level (<25%), indicating potential nonconformity with the test instructions. No individual of the final analysis sample scored below chance level nor did study documentation indicate irregularities regarding task completion.

### Statistical Analysis

#### Dataset Preparation

Firstly, the analysis sample was stratified into sex (male/female), age [20–29 years, 30–39 years, 40–49 years, 50–59 years, and 60 years and older (60+)] and education (≤10 years of school; >10 years of school) groups providing a basic data structure for the calculation of RMET standard scores. Since these standard scores should be applicable for German-speaking subjects, it was necessary that the distribution characteristics of the study sample match the German population regarding the above-mentioned characteristics. As this was not the case *per se*, weighting factors were computed based on German population data published in the “Mikrozensus 2014” study (Statistisches Bundesamt Wiesbaden, 2018). The weighting factors were derived by aligning the number of individuals of the Mikrozensus 2014 age-sex-education subsamples with the number of individuals of the corresponding subsamples of the current study. Weighting factors were then multiplied group-wise with the number of individuals of the study dataset resulting in an overlap with the German population characteristics. See Table 1 for a detailed overview about weighting factor extraction and application to the study dataset.

**Table 1:** Number of participants per group in the German reference population (Mikrozensus 2014; *N* = 63,292), the LIFE adult study (*N* = 966), and the weighted analyses sample (LIFE^*^, *N* = 966).

**Sex**	**Education**	**N Mikrozensus (%)**	***N* LIFE (%)**	**Weighting factor**	*N* LIFE*
**A. Age group: 20–29 years**					
Male	≤10 y	2437 (3.9)	12 (1.2)	3.1	37
	>10 y	2156 (3.4)	30 (3.1)	1.1	33
Female	≤10 y	2043 (3.2)	3 (0.3)	10.4	31
	>10 y	2388 (3.8)	15 (1.6)	2.4	36
Total	≤10 y	4480 (7.1)	15 (1.6)	4.6	68
	>10 y	4544 (7.2)	45 (4.7)	1.5	69
**B. Age group: 30–39 years**					
Male	≤10 y	2711 (4.3)	20 (2.1)	2.1	41
	>10 y	2038 (3.2)	38 (4)	0.8	31
Female	≤10 y	2518 (4)	9 (0.9)	4.3	38
	>10 y	2186 (3.5)	24 (2.5)	1.4	33
Total	≤10 y	5229 (8.3)	29 (3)	2.8	80
	>10 y	4224 (6.7)	62 (6.4)	1	64
**C. Age group: 40–49 years**					
Male	≤10 y	3809 (6)	58 (6)	1	58
	>10 y	2076 (3.3)	27 (2.8)	1.2	32
Female	≤10 y	3800 (6)	44 (4.6)	1.3	58
	>10 y	1908 (3)	20 (2.1)	1.5	29
Total	≤10 y	7609 (12)	102 (10.6)	1.1	116
	>10 y	3984 (6.3)	47 (4.9)	1.3	61
**D. Age group: 50–59 years**					
Male	≤10 y	4075 (6.4)	28 (2.9)	2.2	62
	>10 y	1800 (2.8)	18 (1.9)	1.5	27
Female	≤10 y	4376 (6.9)	27 (2.8)	2.5	67
	>10 y	1556 (2.5)	9 (0.9)	2.6	24
Total	≤10 y	8451(13.4)	55 (5.7)	2.3	129
	>10 y	3356 (5.3)	27 (2.8)	1.9	51
**E. Age group: ≥60 years**					
Male	≤10 y	7281 (11.5)	201 (20.8)	0.6	111
	>10 y	2349 (3.7)	138 (14.3)	0.3	36
Female	≤10 y	10351 (16.4)	176 (18.2)	0.9	158
	>10 y	1434 (2.3)	69 (7.1)	0.3	22
Total	≤10 y	17632 (27.9)	377 (39)	0.7	269
	>10 y	3783 (6)	207 (21.4)	0.3	58

Panels A–E show group-specific absolute number of participants per group in the German reference population (Mikrozensus 2014) and the LIFE adult study. Relative numbers are presented in parenthesis. Weighting factors are calculated as: relative number (Mikrozensus)/relative number (LIFE). In a last step, weighting factors are multiplied with the original number of participants from the LIFE adult study resulting in the final distribution of the current study's analysis sample (N LIFE*).

#### RMET Standard Score Computation

This computation is performed on the aligned dataset (i.e., where weighting factors were applied to match the German population according to age, sex, education). Age-specific frequency distributions of correct responses in the RMET for men and women have been used. For each frequency distribution, percentile ranks were computed as $PR_{v}=\frac{freq_{cum}(x_{v})}{N}*100$ (Moosbrugger and Kelava, 2012, p. 176 et seqq.). The term *freq*_*cum*_(*x*_*v*_) defines the accumulated frequencies up to (and including) the score of interest (i.e., RMET accuracy score); *N* defines the number of participants within the (sub-) sample. The percentage rank informs about an individual's accuracy in the RMET within the reference group. It is defined as the relative number (percentage) of scores within the frequency distribution equal to or lower than it. Percentile ranks were computed separately for men and women of five age groups (20–29, 30–39, 40–49, 50–59, 60+).

## Results

**Figure 2:**
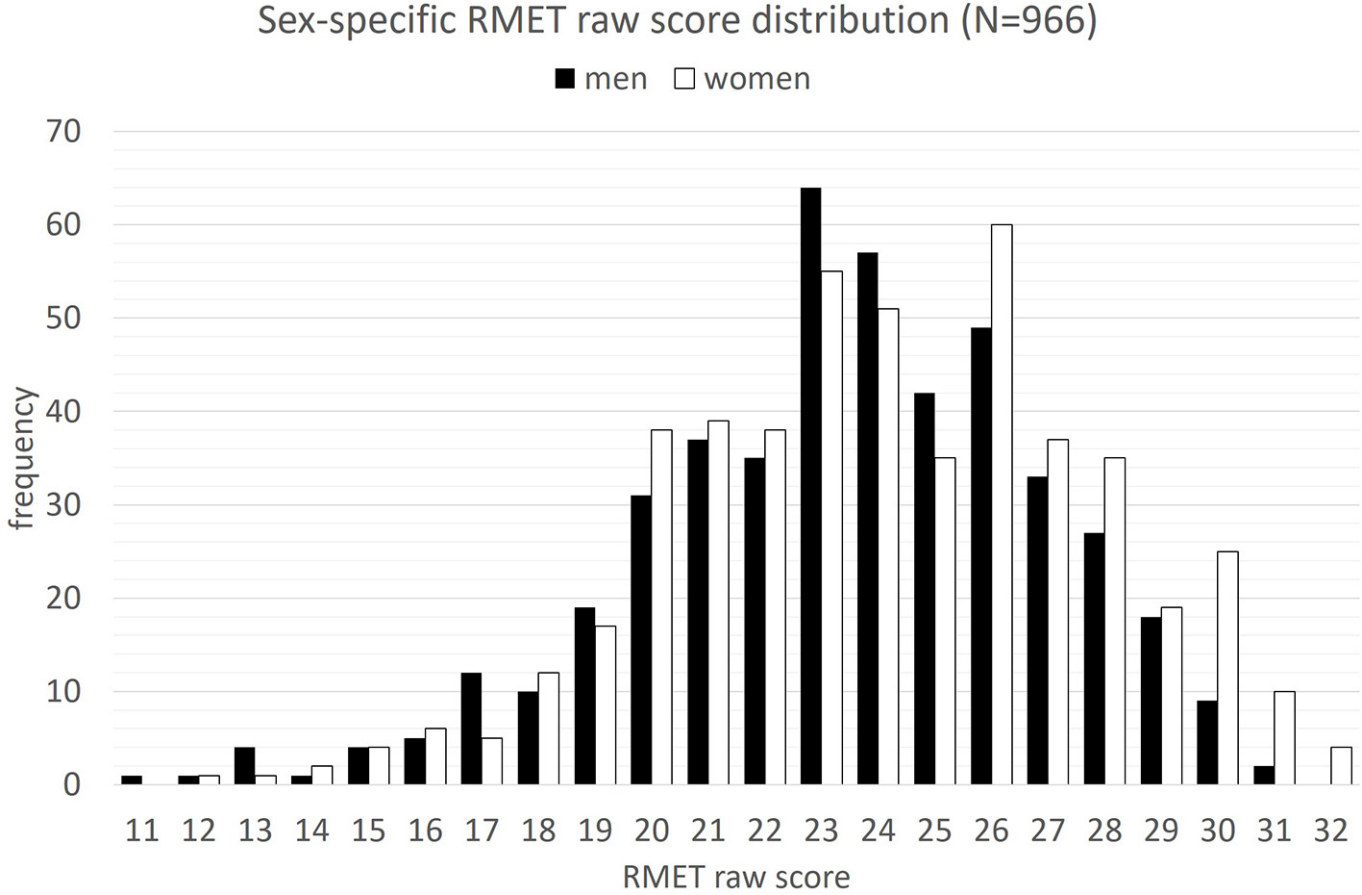
Sex-specific frequency distribution of correct responses in the *Reading the Mind in the Eyes Test (RMET)* across the standardization sample (*N* = 966).

The sample comprises 966 adults (469 male) aged 20–79 (*M* = 50.7, *SD* = 16.2; see Table 2). Descriptive results suggest a trend toward an inverse relation between RMET accuracy rates and age. Also, females scored slightly higher than males on a descriptive level. The sex-specific RMET raw score distribution across the sample is additionally presented in Figure 2. Although women scored on the top level (max accuracy score = 32), both men and women presented identical accuracy spans (20 scores).

**Table 2:** Sociodemographic characteristics of the standardization sample (*N* = 966) and corresponding RMET scores (*M, SD*).

		***N***	**%**	**RMET total correct *M* (*SD*)**
Age (years)	*M* = 50.7	966	100	
	*SD* = 16.2			
	range: 20–79			
Age groups	20–29	138	14.2	26.0 (3.2)
	30–39	144	14.9	24.7 (3.2)
	40–49	177	18.3	24.1 (3.3)
	50–59	180	18.7	23.4 (3.2)
	60+	327	33.8	22.4 (3.8)
Sex	Male	469	48.6	23.4 (3.6)
	Female	497	51.4	24.1 (3.7)
Education	≤10 years	662	68.6	23.3 (3.7)
	>10 years	304	31.4	24.8 (3.4)

*N, sample size; M, mean; RMET, Reading the Mind in the Eyes Test; SD, standard deviation*.

Persons with higher education presented better RMET performance compared to persons with <10 years of school (Table 2). An one-way ANOVA predicting RMET accuracy from age-group (5), sex (2) and education (2) was computed [*F*_(17;945)_ = 9.992, *p* < 0.001; *R*^2^=13.6]. Main effects of the factors age [*F*_(4;945)_ = 24.452, *p* < 0.001), sex [*F*_(1;945)_ = 8.727, *p* < 0.001], and education [*F*_(1;945)_ = 6.571, *p* < 0.001) were found.

Normative scores are shown in Table 3. Here, ranked raw test scores were monotonously transformed into percentile ranks within a specific age and sex group (see again Table 1 for information on weighting factor extraction and application to the study dataset). The maximum scores within an age group are comparable between men and women. Regarding age, older age groups present a wider scoring range compared to younger age groups. For older men/women, lower accuracy rates may therefore be defined as age-appropriate, while an identical score may be classified as “below average” in a younger individual. For instance, a number of 20 correctly identified items corresponds to a percentile rank of 12 in a man younger than 30 (below average), while performance is on average for a man in his 60's with an identical score. Note, that for certain raw scores no corresponding percentage rank could be calculated.

**Table 3:** Age- and sex-specific percentile ranks corresponding to number of correct responses in the *Reading the Mind in the Eyes Test (RMET)*.

**Sex**	**Men**					**Women**				
Age group (years)	20–29	30–39	40–49	50–59	60+	20–29	30–39	40–49	50–59	60+
N	70	72	90	90	147	68	72	87	91	180
**RMET raw score**	**Percentile rank**									
≥32						98				
31						96	98			
30	98	98			99	89			97	99
29	92	94	98		98	70	96	92		97
28	84	91	94	95	97	66	82	87	94	95
27	72	88	85	91	92	47	74	80		92
26	63	77	78	82	89	40	56	74	88	85
25	46	58	72	73	82	33	47	51	69	79
24	41	50	61	63	72	29		42	63	70
23	34	37	47	53	58	19	41	32	47	58
22	23	24	32		49	15		21	41	46
21		15	25	30	38		19	16	33	36
20	12	9	20	15	29		15	10	19	26
19		6	12	12	21		5		11	17
18			10	6	15			3	5	13
17		1	8	4	11			1		9
16			3		8					7
15			1	2	6					4
14					5					2
13					4					1
≤12					1					

*N, sample size*.

## Discussion

In this study, we computed age- and sex-standardized scores for the German version of the RMET based on a population-based sample including 966 healthy adults. Scores were weighted to match the demographic characteristics of the German population. This is the first study publishing standard scores for the frequently used RMET. The RMET assesses a specific aspect of ToM, i.e., the ability to identify complex mental states from gaze. Based on the guidelines for the evaluation of cognitive performance in the framework of NCD introduced by the DSM-5 (American Psychiatric Association, 2013), we provide age- and sex-specific standard scores improving the reliable distinction between typical and atypical test performance for a potential application in the clinical diagnostics of social cognitive abilities.

Notably, this study goes beyond clinical aspects of socio-cognitive impairment, as it addresses the ability to identify mental states from gaze in healthy adults across the adult age range. With this, it is linked to the concept of lifespan development (Baltes, 1987). Age may affect social cognitive abilities differently in men and women, but recent findings regarding the RMET are contradictory (e.g., Kirkland et al., 2013). Our results suggest slight performance differences between men and women, justifying the computation of specific standard scores. In both men and women, performance is reduced with older age.

Besides the remarkably large number of individuals used for standardization and the wide age-span covered, data was weighted according to age, sex, and education status to match the population characteristics of Germany. With this, standard scores are not limited to the current sample, but may be generally applied across German-speaking areas. Notably, rank transformation does not require normal distribution, but the shape of the distribution function informs about the group-specific frequency of RMET scores (cf. Moosbrugger and Kelava, 2012). This may be helpful for the interpretation of individual performance in relation to the standardization sample. Importantly, sections within the frequency distribution where scores are denser may lead to an overestimation of performance differences, while performance differences in less dense sections may be underestimated.

By addressing these points, our study contributes essentially to a systematic assessment of socio-cognitive abilities, which is an important aspect in clinical diagnostics of psychiatric, neurological, and neurodegenerative diseases associated with ToM impairment. Moreover, defining the range of extraordinary test performance enables to identify persons with distinct social cognitive abilities, which may serve as an important personal resource and cognitive reserve deferring cognitive decline.

### Limitations and Implications for Application in Diagnostic Settings

Although this study was carefully conducted, some limitations need to be considered. Firstly, the stratification of the current sample according to sex, age, and education as a basic structure for standard score computation may seem arbitrary. However, it is common sense in the field of neuropsychology that basic demographic factors potentially impact cognitive performance. The ANOVA results additionally justify this approach. Additionally, it enables comparability with other studies and facilitates application in clinical settings. The binary coding of sex/gender information may be regarded conventional as it neglects persons with differing sexual identity and personal self-concept potentially influencing socio-cognitive functioning (e.g., Kung, 2020). Thus, standard scores provided here may not be applicable in these cases. In future, it may be helpful to consider concepts beyond the classical sex/gender dimorphism picturing sexual diversity in populations. Of note, information on sex/gender was obtained directly from the resident's registration office of the city of Leipzig, and, additionally, via self-report. All participants showed agreement on both factors (see also Kynast et al., 2020). Furthermore, other factors such as environment, culture and experience may drive (social) cognitive performance beyond sex/gender (see Jäncke, 2018 for review; but also Dotson and Duarte, 2020 for the importance of sex/gender in neuroscience). Secondly, education was dichotomized based on years of formal education and graduation diploma for practicability reasons. Yet, tertiary education or further professional training was not considered for standardization although it possibly modulates RMET performance. This effect on mental state attribution remains subject to future research. Thirdly, numbers of subjects differ in the several groups. Here, the relatively large age group 60+ spans two decades of adults instead of being separated into one group for each decade (60–69, 70–79). This was done to provide an appropriate number of elderly individuals with higher education (cf. Table 1) for standardization, since formal scholastic education of more than 10 years was unusual among persons growing up from between the late 1930s and early 1950s in Germany, which can be regarded as a cohort effect. Although a broad definition of this age group may thus be justified and the application of weighting factors ensures matching distribution characteristics with the German population, it must be considered that the individual test performance of a person with advanced age may be characterized less precisely under these conditions. The original study distribution did not exactly match the characteristic proportions regarding age, sex and education in the German population, which is a limitation of the study. We tried to overcome this issue by the application of weighting factors. We based this on the latest available distribution data (Statistisches Bundesamt Wiesbaden, 2018). With more than 950 individuals, the sample size can be regarded appropriate for standardization. Yet, the subsamples used for the computation of sex-specific standard scores contain <100 individuals per group and it must be noted that in those groups not all percentage ranks may correspond to distinct RMET raw scores. Hence, our data corresponds with previous studies since again, the test does not have a ceiling effect (Pardini and Nichelli, 2009), supporting its eligibility for clinical application.

However, it must be critically addressed that the standard scores provided here are not sufficient for the diagnosis of psychiatric disorders, neurological or neurodegenerative diseases but rather indicate difficulties in a specific aspect of ToM, i.e., the ability to identify mental states from gaze. Deficits obtained in this test must be verified by subsequent, in-depth diagnostic procedures. Furthermore, RMET performance may be modulated by other individual factors that have not been considered here. For instance, it has been shown that RMET performance is related to verbal intelligence, but also other cognitive abilities (Ahmed and Miller, 2011; Peterson and Miller, 2012; Baker et al., 2014; Cabinio et al., 2015; Kynast et al., 2018, 2020) that possibly enhance or decrease mindreading accuracy. Moreover, test performance may be influenced by the RMET's stimulus characteristics (Kynast and Schroeter, 2018). Thus, these factors should be additionally assessed and considered in diagnostic settings.

Overall, the RMET, with all its strengths and limitations, can thus be a useful assessment instrument indicating socio-cognitive deficits in specific psychiatric disorders and neurological diseases. In a more resource-oriented view this test may also be used to detect individual strengths in this cognitive domain that might improve the use of therapeutic interventions or be used in professional settings (e.g., identify applicants with extraordinary skills in specific sectors such as health care or elderly care).

### Conclusion

In conclusion, this is the first study providing standard scores for the ability to identify mental states from gaze. They may be used for the detection of socio-cognitive deficits in clinical practice, e.g., in the context of dementia, other neurological diseases and psychiatric disorders, or the assessment of socio-cognitive resources that may be strengthened by prevention or rehabilitation strategies. This large sample of healthy adults was weighted to match the distribution characteristics regarding age, sex, and education of the German population, enabling the application of RMET standard scores to German-speaking areas.

## Data Availability Statement

The data analyzed in this study is subject to the following licenses/restrictions: Data can be requested upon reasonable request. Requests to access these datasets should be directed to Matthias L. Schroeter, schroet@cbs.mpg.de.

## Ethics Statement

The studies involving human participants were reviewed and approved by Ethics Committee University of Leipzig. The patients/participants provided their written informed consent to participate in this study.

## Author Contributions

JK planned and conducted statistical analyses, wrote the first draft of the manuscript including creation of the figures, and modified all subsequent drafts. MP was responsible for data quality management and contributed substantially to all drafts of the manuscript. EQ planned the study, assessed and processed data, and substantially contributed to all drafts of the paper. AH contributed substantially to the interpretation of the results and to the manuscript. AV designed the study, interpreted results, and reviewed the final draft of the manuscript. MS designed the study, supervised data acquisition and analyses, substantially contributed to the interpretation of the results, and made substantial modifications to all drafts of the manuscript. All authors contributed to the article and approved the submitted version.

## Conflict of Interest

The authors declare that the research was conducted in the absence of any commercial or financial relationships that could be construed as a potential conflict of interest.
